# Conversational Agents for Health and Well-being Across the Life Course: Protocol for an Evidence Map

**DOI:** 10.2196/26680

**Published:** 2021-09-17

**Authors:** Mara Pereira Guerreiro, Leonardo Angelini, Helga Rafael Henriques, Mira El Kamali, Cristina Baixinho, João Balsa, Isa Brito Félix, Omar Abou Khaled, Maria Beatriz Carmo, Ana Paula Cláudio, Maurizio Caon, Karl Daher, Bruno Alexandre, Mafalda Padinha, Elena Mugellini

**Affiliations:** 1 Nursing Research, Innovation and Development Centre of Lisbon Nursing School of Lisbon Lisbon Portugal; 2 Centro de Investigação Interdisciplinar Egas Moniz Instituto Universitário Egas Moniz Monte de Caparica Portugal; 3 University of Applied Sciences and Arts Western Switzerland Fribourg Switzerland; 4 CiTechare Leiria Portugal; 5 LASIGE Faculdade de Ciências Universidade de Lisboa Lisbon Portugal; 6 Nursing School of Lisbon Lisbon Portugal; 7 Instituto Universitário Egas Moniz Monte de Caparica Portugal

**Keywords:** artificial intelligence, conversational agent, chatbot, virtual assistant, relational agent, virtual humans, e-coach, intervention, health, well-being

## Abstract

**Background:**

Conversational agents, which we defined as computer programs that are designed to simulate two-way human conversation by using language and are potentially supplemented with nonlanguage modalities, offer promising avenues for health interventions for different populations across the life course. There is a lack of open-access and user-friendly resources for identifying research trends and gaps and pinpointing expertise across international centers.

**Objective:**

Our aim is to provide an overview of all relevant evidence on conversational agents for health and well-being across the life course. Specifically, our objectives are to identify, categorize, and synthesize—through visual formats and a searchable database—primary studies and reviews in this research field.

**Methods:**

An evidence map was selected as the type of literature review to be conducted, as it optimally corresponded to our aim. We systematically searched 8 databases (MEDLINE; CINAHL; Web of Science; Scopus; the Cochrane, ACM, IEEE, and Joanna Briggs Institute databases; and Google Scholar). We will perform backward citation searching on all included studies. The first stage of a double-stage screening procedure, which was based on abstracts and titles only, was conducted by using predetermined eligibility criteria for primary studies and reviews. An operational screening procedure was developed for streamlined and consistent screening across the team. Double data extraction will be performed with previously piloted data collection forms. We will appraise systematic reviews by using A Measurement Tool to Assess Systematic Reviews (AMSTAR) 2. Primary studies and reviews will be assessed separately in the analysis. Data will be synthesized through descriptive statistics, bivariate statistics, and subgroup analysis (if appropriate) and through high-level maps such as scatter and bubble charts. The development of the searchable database will be informed by the research questions and data extraction forms.

**Results:**

As of April 2021, the literature search in the eight databases was concluded, yielding a total of 16,351 records. The first stage of screening, which was based on abstracts and titles only, resulted in the selection of 1282 records of primary studies and 151 records of reviews. These will be subjected to second-stage screening. A glossary with operational definitions for supporting the study selection and data extraction stages was drafted. The anticipated completion date is October 2021.

**Conclusions:**

Our wider definition of a conversational agent and the broad scope of our evidence map will explicate trends and gaps in this field of research. Additionally, our evidence map and searchable database of studies will help researchers to avoid fragmented research efforts and wasteful redundancies. Finally, as part of the Harnessing the Power of Conversational e-Coaches for Health and Well-being Through Swiss-Portuguese Collaboration project, our work will also inform the development of an international taxonomy on conversational agents for health and well-being, thereby contributing to terminology standardization and categorization.

**International Registered Report Identifier (IRRID):**

DERR1-10.2196/26680

## Introduction

In 2016, noncommunicable diseases (NCDs) accounted for 40.5 million deaths worldwide, which corresponded to 71% of deaths worldwide. The top 4 NCDs are cardiovascular diseases, cancers, diabetes, and chronic lung diseases [1]. NCDs can be prevented by adopting a healthy lifestyle. For example, a European multicohort study, which was conducted from 1991 to 2006 and included 116,043 people who were free of major NCDs at baseline, suggested that various healthy lifestyle profiles yield gains in life years without major NCDs, including type 2 diabetes, coronary heart disease, stroke, cancer, asthma, and chronic obstructive pulmonary disease [2].

Changing and sustaining health behaviors, which are integral to both the prevention and self-management of NCDs, are known to be challenging and resource intensive [3]. Digitalization and automation remove time and place restrictions, thereby broadening access to lifestyle and self-management interventions in a potentially cost-effective manner. For example, full economic evaluations of interventions that use the internet, mobile devices, or computers for the prevention and control of type 2 diabetes have demonstrated high cost-effectiveness, even though they were not fully automated [4].

The European Blueprint on Digital Transformation of Health and Care for the Ageing Society reflects a common vision that key stakeholders have on the role of innovation in changing health and care provision among older populations [5]. The priority topics encompassed by this policy vision are disease prevention, personalized health and care, and digital tools for citizen empowerment and person-centered care. A total of 12 blueprint personas have been created based on the health and care needs of people across the life course, ranging from children to persons aged ≥80 years [5].

Digital technology that mimics human communication is suitable for different age populations and populations with different literacy levels and is arguably more engaging for long-term use. A scan of this landscape has revealed a considerable body of scientific literature, although no agreements have emerged on the definition of so-called conversational agents. For instance, some authors consider conversational agents to be software capable of natural language processing [6]. However, others have used broader definitions that encompass agents that use predefined text options as inputs but exclude embodied agents that use nonverbal communication [7].

For the purpose of our review, we defined conversational agents as computer programs that are designed to simulate two-way human conversation by using language (speech or text) and are potentially supplemented with nonlanguage modalities. We believe that conversational agents for health and well-being should be further characterized according to the health intervention (eg, target population, design, the entity on which the intervention is carried out, and duration), the agent (eg, embodiment, role, and delivery channel), and the conversation (eg, input and output options, dialogue engine, and sentiment detection). We derived these ideas from literature [7-11], the international classification of health interventions [12], our interdisciplinary experience [13-16], and discussions within the research team.

This study is part of the Harnessing the Power of Conversational e-Coaches for Health and Well-being Through Swiss-Portuguese Collaboration (eCCo) project [17], which encompasses an evidence map and the subsequent development of an international taxonomy on conversational agents for health and well-being via a scientific consensus method that will be informed by our literature review. In addition to this purpose, the evidence map independently serves a much-needed research endeavor—fostering collaboration in the field through an open-access resource. In their review on conversational interfaces for health, Xing et al [18] highlighted the need to improve collaboration among stakeholders in research and patent activities. To our knowledge, there is no open, searchable database on conversational agents for health—a resource that could foster collaboration by pinpointing expertise across international centers and networks. Such collaboration can help with tackling research fragmentation and duplication.

Our aim is to provide an overview of all relevant evidence on conversational agents for health and well-being across the life course. Specifically, our objectives are to identify, categorize, and synthesize primary studies and reviews on this topic by focusing on the following research questions:

- What is the nature of literature on conversational agents for health and well-being (eg, information source, research group, and study characteristics)?
- What are the characteristics of health interventions based on conversational agents (eg, setting, target population, intervention target, duration, and frequency)?
- What are the characteristics of the automated conversations conducted in health interventions (eg, interaction input and output and dialogue engine)?
- What are the characteristics of the agents used in health interventions (eg, embodiment, emotions, role, and delivery channel)?

## Methods

### Evidence Map

An evidence map is “a systematic search of a broad field to identify gaps in knowledge and/or future research needs that presents results in a user-friendly format, often a visual figure or graph, or a searchable database” [19]. These reviews typically encompass different types of studies, such as reviews and primary studies. They rely on a systematic search strategy, conducting screening based on explicit eligibility criteria, and conducting data extraction in a structured format. Critical appraisal may be performed, but it is not required [19].

### Searching

#### Keyword Selection and Initial Database Query

To comprehensively identify relevant keywords, we resorted to using a purposive sample of 13 literature reviews [6,7,9,10,18,20-29] and a review protocol [30] related to conversational agents for health and well-being. A total of 318 keywords were extracted. The removal of duplicates resulted in 220 keywords.

Keywords were categorized into tentative domains and tested in MEDLINE; we resorted to using PubMed as the interface. The search process was documented and iteratively optimized [31] to yield a compromise between feasibility and completeness. This led to the choice of using the following two final keyword domains: K1 (variations of conversational agent–related terms) and K2 (variations of health-related and well-being–related terms). We expanded the K1 domain by including all variations and combinations of the terms *agent* (ie, *bot*, *robot*, *assistant*, *coach*, *companion*, *system*, *avatar*, and *entity program*) and *conversational* (ie, *talking*, *voice*, *communication*, *social*, *dialogue*, and *utterance*). We also included terms in the K1 domain related to popular commercial conversational agents, such as *Google Home*, *Google Assistant*, *Cortana*, *Alexa*, and *Siri*.

The search strategy encompassed (when applicable) plural forms of keywords and variations at the end of keywords, which were indicated with the wildcard asterisk (ie, “*”). We accounted for variations in the middle of phrases and hyphenation by using similar vocabulary (eg, *talk bot* and *talkative bot*, *ecoach* and *e-coach*, etc). We ended up with 265 keywords for the K1 domain and 13 keywords for the K2 domain.

There was ambiguity between the terms *Amazon Alexa assistant* and *alexa fluor compounds*. Therefore, a third keyword domain was developed (K3), which consisted of variations of *alexa fluor compounds* to be excluded from the search query. This domain limited the number of irrelevant results through the use of database syntax.

The search string for the search conducted on MEDLINE via PubMed is depicted in Multimedia Appendix 1. The search was restricted to titles and abstracts only.

#### Database Selection

Initially, we listed the data sources used by the aforementioned sample of studies [6,7,9,10,18,20-30]. This led to a set of the following nine potentially useful scientific literature databases: MEDLINE; CINAHL; Web of Science; Scopus; the Cochrane, ACM, IEEE, and Joanna Briggs Institute (JBI) databases; and Google Scholar. These were then analyzed in terms of their coverage and suitability.

Both ACM and IEEE publish computer science conference proceedings, and these were relevant to our evidence map and supplemented our health data sources. Although Scopus and Web of Science cover most journal publications from ACM and IEEE, their indexing of conference proceedings is poorer [32]. Therefore, from a coverage standpoint, it would make sense to retain these databases.

In terms of the search quality of the data sources, the work of Gusenbauer and Haddaway [33] endorsed the choice of using MEDLINE, CINAHL, Web of Science, Scopus, Cochrane, and ACM. These authors did not consider Google Scholar to be an appropriate principal search system, since it does not allow for the use of Boolean queries and does not provide consistent results over time [33].

After taking both coverage and search qualities into account, we decided to retain Google Scholar and ACM in the final list of databases. When compared to Scopus and Web of Science, Google Scholar is still the most far-reaching source [34]. Scopus and Web of Science exhibit indexing lags and may miss the latest publications, unlike Google Scholar [35,36]. However, we discarded IEEE from the list on the grounds of its limited search capabilities, as we were unable to search this database for all the proposed keywords.

The decision to exclude grey literature was dictated by our available resources. A definition of grey literature was put forward during the 1997 International Conference on Grey Literature in Luxembourg and was expanded in 2004 (in New York) as “information produced on all levels of government, academics, business and industry in print and electronic formats, but which is not controlled by commercial publishers, i.e. where publishing is not the primary activity of the producing body” [37]. Recently, Garousi et al [38] proposed a wider definition for grey literature in the software engineering field and grouped grey literature into 3 tiers according to expertise (ie, the established knowledge of the content producer) and outlet control (ie, content production in conformance with explicit and transparent criteria). The tiers encompass content from blogs, tweets, and news articles; presentations, and government reports. Regardless of the definition, grey literature would add to the predictably extensive amount of formal literature and was deemed to be of uncertain value in light of the review’s aim. Grey literature would also require additional resources for analysis.

#### Testing the Query on the Remaining Databases

The PubMed search query was used in CINAHL (via EBSCO [Elton B. Stephens Company]); Web of Science; Scopus; and the Cochrane (via EBSCO), ACM, and JBI databases; minor adjustments were made [31]. The restriction to a title and abstract search was maintained in these databases, with the exception of the JBI database, which does not allow for such searches.

The Google Scholar query, which is presented in Multimedia Appendix 1, comprised short forms of the K1 and K2 domain terms. This search was limited to literature published from 2020 onward and those without patents and citations. The search results were sorted by relevance. As recommended by Haddaway et al [39], we retrieved only the first 300 results.

#### Citation Searching

We will conduct a backward citation search by manually searching the reference lists of all articles included in the evidence map. Forward citation searching by using a citation index to identify studies that cite included articles [40] was deemed unfeasible in light of the project resources.

### Selection of Studies

Rayyan (Rayyan Systems Inc)—a collaborative web application—was used to streamline the selection of studies [41]. It supports the screening and coding of studies, documents reviewers’ decisions by using tags, and allows for the organization of records via filters.

Teams of 2 researchers will independently screen retrieved records by using predetermined eligibility criteria for primary studies and reviews, which are detailed in Textboxes 1 and 2. Meeting all of the inclusion criteria will be a requirement for an article to be selected for the evidence map. Selected articles must also not meet any of the exclusion criteria.

**Eligibility criteria for primary studies on conversational agents for health and well-being.**

**Inclusion criteria**

- Primary studies that focus on persons of all ages regardless of their health status
- Presenting a computer program that is able to simulate two-way human conversation for a health-related purpose or general well-being–related purpose by using language (speech or text) and is potentially supplemented with nonlanguage modalities, regardless of the input and output options
- Reporting the design, development, evaluation, or implementation of conversational agents regardless of the involvement of human users and study design

**Exclusion criteria**

- Articles focused solely on caregivers, health care professionals, or the education of health care professionals or students
- Articles that do not concomitantly report information on the following three components: the health intervention, the agent, and the conversational capabilities (eg, articles focused on individual features only, such as speech recognition)
- Agents without automated conversational capabilities (eg, Wizard of Oz tool)
- Press articles
- Unavailable full text
- Articles written in languages other than English, Italian, French, Portuguese, and Spanish
- Commentaries, opinion papers, position papers, study protocols, or any article not presenting primary research (eg, discussing the intention to develop a conversational agent)
- Conference abstracts

**Eligibility criteria for reviews on conversational agents for health and well-being.**

**Inclusion criteria**

- All review designs that focus on primary studies on human participants regardless of their health status
- Reviews comprised of studies that present a computer program that is able to simulate human conversation for a health-related purpose or general well-being–related purpose by using language and is potentially supplemented with nonlanguage modalities, regardless of the input and output options
- Reporting the design, development, evaluation, implementation, or funding of conversational agents regardless of the involvement of human users and study design

**Exclusion criteria**

- Reviews that include conversational agents for the education of health care professionals or students or another nonhealth purpose
- Reviews including studies on nonconversational agents or those without automated conversational capabilities (eg, Wizard of Oz tool)
- Review protocols
- Unavailable full text
- Articles written in languages other than English, Italian, French, Portuguese, and Spanish

The screening procedure was piloted at the commencement of this stage; we used a set of primary studies and reviews. In the first stage, we reviewed the titles and abstracts of retrieved records. Discrepancies in inclusion and exclusion decisions were resolved by a third reviewer. In the second stage we will focus on the full-text review of the records selected in the first stage. Discrepancies in inclusion and exclusion decisions at this stage will be resolved by discussion between the reviewer pairs, and if a consensus is not reached, a third researcher will be involved.

### Data Extraction

Data collection forms for primary studies and reviews were designed using Microsoft Excel spreadsheets. In addition to general information such as article ID numbers (unique identifiers), titles, and aims, we will extract information related to our research questions. Moreover, we will extract definitions of conversational agents if they are provided. The forms will be piloted with a set of primary studies and reviews to ensure that they capture relevant information comprehensively. Multimedia Appendix 2 presents variables that were preliminarily included in the Excel spreadsheet.

Teams of 2 researchers will independently extract data from included records. Potential discrepancies will be resolved via consultation with a third researcher.

### Critical Appraisal

As previously explained, critical appraisal is recommended for evidence maps but is not mandatory [19]. Therefore, based on project resources, we will conduct the critical appraisal of systematic reviews, but this will not be done for the anticipated large number of primary studies. A Measurement Tool to Assess Systematic Reviews (AMSTAR) 2—a revised 16-item version of AMSTAR—will be used to evaluate the quality of included systematic reviews [42]. AMSTAR 2 takes longer to apply than AMSTAR; however, both have higher levels of interrater reliability compared to those of similar tools, such as the Risk of Bias Assessment Tool for Systematic Reviews (ROBIS) [43]. Compared to the ROBIS, AMSTAR 2 is easier to apply. Further, guidance on using AMSTAR 2 is clearer and simpler, which promotes its use by nonexperienced reviewers [44].

### Data Synthesis

We will assess primary and secondary studies separately in the analysis. Each data set will be subjected to a descriptive analysis to summarize the characteristics of included studies. This will be guided by our research questions. The bivariate exploration of data will be conducted, as appropriate. Subgroup analyses will be conducted, if feasible.

High-level maps, such as scatter charts and bubble charts, will be used to depict results and illustrate research trends and gaps. The development of the searchable database will be informed by the research questions and data extraction forms.

## Results

**Figure 1:**
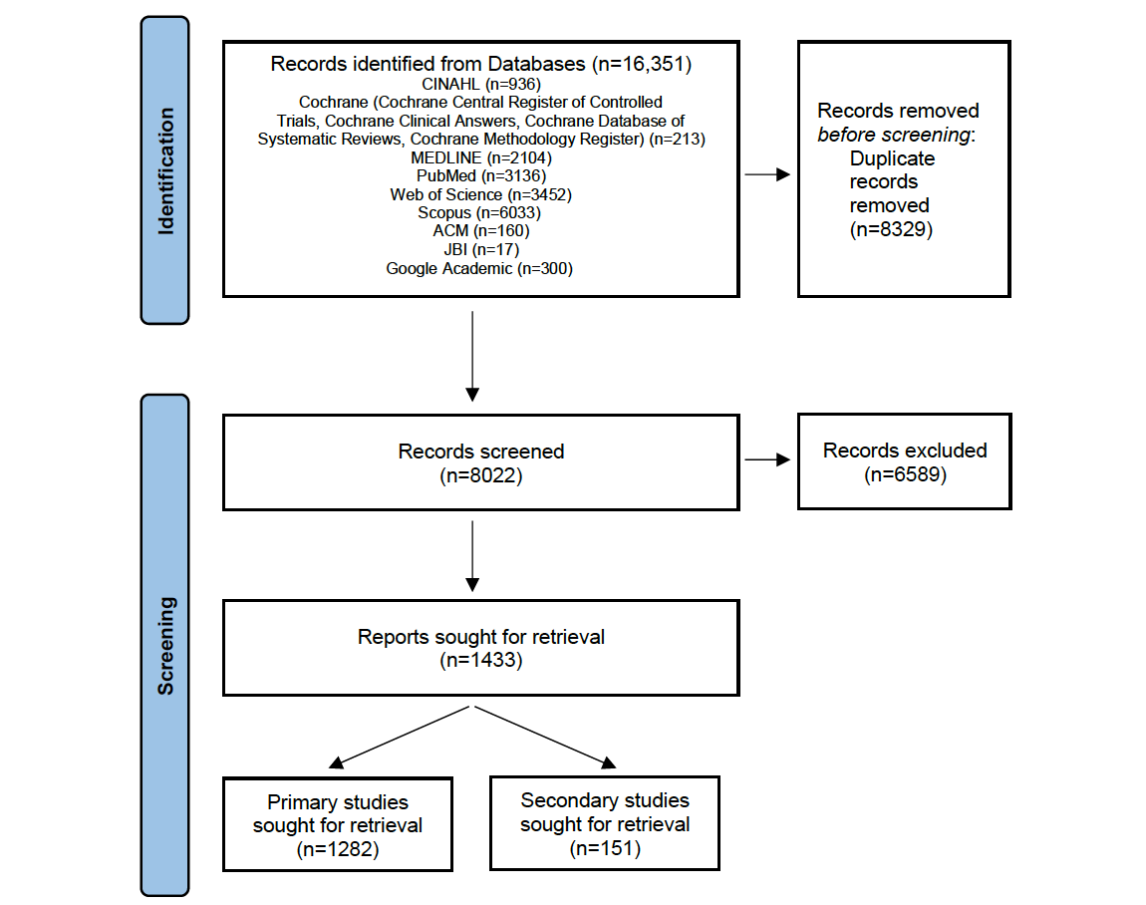
A PRISMA (Preferred Reporting Items for Systematic Reviews and Meta-Analyses) flowchart of the literature search and selection procedures (first stage of screening) [49]. JBI: Joanna Briggs Institute.

The literature search in MEDLINE; CINAHL; Web of Science; Scopus; the Cochrane, ACM, IEEE, and JBI databases; and Google Scholar was conducted between November 11 and November 19, 2020. A total of 16,351 records were identified and exported to Rayyan. The removal of duplicates yielded 8022 records, which were subjected to screening (Figure 1).

As of April 2021, we produced an operational procedure to support screening (aided by Rayyan) to ensure consistency and reduce the amount of errors. We also drafted a glossary with operational definitions to support the study selection and data extraction procedures. This is regarded as a living document, which will be updated as our work progresses (Multimedia Appendix 3; the glossary is currently based on 5 publications [7,45-48]). Moreover, we concluded the first the stage of screening, as depicted in Figure 1.

## Discussion

Our work aims to provide an overview of all relevant evidence on conversational agents for health and well-being across the life course. The evidence and gap map is a type of literature review that is particularly suited for this aim. As White et al [50] put it, “[evidence and gap maps] usually show what evidence is there, not what the evidence says.” By following the same systematic approach as a systematic review, we aim to offer a wider picture of the research landscape by including both primary studies and meta-research. To the best of our knowledge, published reviews on conversational agents, such as those of Car et al [7] and Chattopadhyay et al [27], have only included primary studies. Therefore, we add to current knowledge by extending the scope of existing reviews. As for other evidence maps, the novelty of our evidence synthesis comes from its breadth (ie, summarizing all reviews and primary studies on the topic without collating effects or effect sizes), while the strength of the scoping reviews comes from their depth (ie, typically a narrower scope and direction of effect) [50]. Another aspect regarding the broader scope of our work is our definition of a conversational agent, which comprises not only chatbots but also agents with physical or web-based embodiments, such as robots or anthropomorphic web-based agents.

The number of reviews that were preliminarily uncovered by our review also suggests that it may appropriate to conduct an umbrella review of meta-research in the future. This will help those aspiring to conduct a review in this field to avoid wasteful redundancies. The number of reviews we uncovered raises the issue of the degree of overlap and the incremental value of these publications. Recently, Tugwell et al [51] elaborated on the replication of systematic reviews. In addition to direct replication, which involves repetition for verifying results, these authors put forward the concept of conceptual replication, in which a research question is broadened or narrowed to ascertain different intervention types, settings, outcomes, or study designs [51]. The data extraction of the included reviews in our work will clarify whether these reviews performed conceptual replication or undesirable repetition, which has been coined as *research waste* [51]. We envisage that the open, searchable database that will be developed in the data synthesis stage will help researchers to avoid future research waste by highlighting published reviews.

Another methodological consideration is the role of bias and its influence on the evidence map results. For instance, we addressed bias in the selection of studies through a multifold procedure [52]. First, we detailed the research questions and the inclusion and exclusion criteria to avoid inconsistent application. Second, we developed a glossary of operational definitions to reduce discrepancies in the interpretation of key terms. Third, per the review protocol, two independent reviewers will screen and extract data, and a third reviewer will be involved when discrepancies cannot be resolved by consensus. Other procedures specified in the protocol include presenting a PRISMA (Preferred Reporting Items for Systematic Reviews and Meta-Analyses) flow diagram for results, pilot testing the several steps of the review method (searching, selection of studies, data extraction and critical appraisal), and having workflows subsumed in the evidence map.

The evidence and gap map relies on a framework for detailing its dimensions, which will be operationalized by using row and column headings [50]. The eCCo project will tackle this issue by pursuing an integrated approach based on the interdependency of its two core activities, as follows: (1) producing an evidence map on conversational agents in health and well-being and (2) consensualizing a taxonomy on the topic. The matrix for the data extraction (Multimedia Appendix 2) was informed by the first draft of this taxonomy, and the evidence map will allow for the identification of international experts who will be involved in the consensus conference. Further, the results of the evidence map will be used to fine-tune the taxonomy draft before the draft is subjected to the scrutiny of international experts. Our research project is in line with the work of Bittner et al [53], who used an empirical-to-conceptual approach for the development of a taxonomy for conversational agents and drew upon a literature review to identify new subsets of objects. We identified a set of taxonomies in the field, albeit none were health specific [8,9]. The foci of all these taxonomies are design options that do not detail the aspects of the health intervention. Guidelines for reporting on digital health interventions [54,55] have recommended the specification of intervention components and modes of delivery (eg, specifying who delivers the intervention, who receives the intervention, how often the intervention is delivered, the intervention duration, the format of the intervention, and the context in which the intervention is delivered). These requirements were considered when first drafting the eCCo taxonomy.

None of the above-mentioned taxonomies used a scientific consensus process to standardize terminology and categories of conversational agents. This is a limitation that we are addressing via the eCCo project.

In spite of its clear strengths, the review will not be without limitations. Integral to evidence maps is the fact that study outcomes are not extracted to ascertain effects. Moreover, the fact that we will not appraise the quality of primary studies means that we cannot pinpoint research gaps for areas with high volumes of potentially poor-quality studies. Another limitation is that research gaps do not necessarily translate to research needs; when prioritizing research needs, one should consider aspects such as relevance and potential impact. Nonetheless, synthesizing evidence on health-focused conversational agents will facilitate the prioritization of strategic research by commissioners.

In addition to fostering collaboration, we envisage that the open, searchable database will also contribute to bridging the translational gap by, for instance, identifying projects with a higher technology readiness level. Such projects can more easily reach the market via partnerships with the business sector.

## Appendix

Multimedia Appendix 1 MEDLINE and Google Scholar search.

Multimedia Appendix 2 Preliminary data extraction form.

Multimedia Appendix 3 Operational definitions.

## Abbreviations

AMSTAR: A Measurement Tool to Assess Systematic Reviews
EBSCO: Elton B. Stephens Company
eCCo: Harnessing the Power of Conversational e-Coaches for Health and Well-being Through Swiss-Portuguese Collaboration
JBI: Joanna Briggs Institute
NCD: noncommunicable disease
PRISMA: Preferred Reporting Items for Systematic Reviews and Meta-Analyses
ROBIS: Risk of Bias Assessment Tool for Systematic Reviews
