# Supplementary material for: Conversational Agents for Health and Well-being Across the Life Course: Protocol for an Evidence Map
Source: JMIR Res Protoc. 2021 Sep 17;10(9):e26680. doi: 10.2196/26680 (PMC8486996; doi:10.2196/26680)
Supplement: Multimedia Appendix 1 [file resprot_v10i9e26680_app1.pdf]

MEDLINE search query via PubMed

Search restricted to title and abstract, performed on 11/11/2020

Domain	Search string
K1 (conversational agent)	(“animated conversational agent*” OR “artificial conversational entit*” OR “artificial conversation entit*” OR “artificial companion*” OR “artificial intelligence chatbot*” OR “artificial intelligent chat agent*” OR “artificial intelligent chatbot*” OR “assistive app*” OR “assistant app*” OR “assistive chat program*” OR “assisting chat program* ” OR “assistant chat program* ”OR “automated chat agent*” OR “automated dialogue system*” OR “assistive social agent*” OR “assisting social agent*”OR “assistant social agent*” OR “assistive social agent*” OR “chat assist*” OR “chat bot*” OR “chatbot program*” OR “chatbot*” OR “chatterbot*” OR “chatterbox*” OR “communicative robot*” OR “communicative agent*” OR “communication robot*” OR “communication agent*” OR “conversation ai” OR “conversation agent*” OR “conversation agent-based system*” OR “conversation assist*” OR “conversation avatar” OR “conversation bot*” OR “conversation robot*” OR “conversation character*” OR “conversation coach*” OR “conversation e-coach*” OR “conversation ecoach*” OR “conversation computer*” OR “conversation humanoid*” OR “conversation interaction*” OR “conversation interface*” OR “conversation partner*” OR “conversation personal assist*” OR “conversation system*” OR “conversation user-interface” OR “conversational ai” OR “conversational agent*” OR “conversational agent-based system*” OR “conversational assist*” OR “conversational avatar*” OR “conversational bot*” OR “conversational robot*” OR “conversational character*” OR “conversational coach*” OR “conversational e-coach*” OR “conversational ecoach*” OR “conversational computer*” OR “conversational humanoid*” OR “conversational interaction*” OR “conversational interface*” OR “conversational partner*” OR “conversational personal assist*” OR “conversational robot*” OR “conversational system*” OR “conversational user-interface*” OR “dialogue system*” OR “eca” OR “embodied conversation agent*” OR “embodied conversation assist*” OR “embodied conversation avatar*” OR “embodied conversational agent*” OR “embodied conversational assist*” OR “embodied conversational avatar*” OR “home dialogue system*” OR “humanlike character*” OR “humanoid agent*” OR “humanoid assist*” OR “humanoid avatar*” OR “humanoid interaction*” OR “humanoid robot*” OR “intelligent conversation assist*” OR “intelligent conversation avatar*” OR “interactive conversation assist*” OR “intelligent conversational assist*” OR “intelligent conversational avatar*” OR “interactive conversational assist*” OR “interactive talk program*” OR “interactive talking program*” OR “interactive voice interface*” OR “interactive agent*” OR “interactive virtual agent*” OR “interface agent*” OR “online chat agent*” OR “online chat program*” OR “multimodal interaction*” OR “persuasive eca” OR “psychological interact*” OR “question-answer system*” OR “question-answers system*” OR “relational agent*” OR “relational assist*” OR “relational avatar*” OR “relational robot*” OR “relational bot*” OR “relation agent*” OR “relation assist*” OR “relation avatar*” OR “relation robot*” OR “relation bot*” OR “smart speaker*” OR “smart bot*” OR “smart robot*” OR “smart assist*” OR “smart avatar*” OR “smart agent*” OR “sociable agent*” OR “sociable agent*” OR “sociable assist*” OR “sociable avatar*” OR “sociable robot*” OR “sociable communicative machine*” OR “social agent*” OR “social agent*” OR “social assist*” OR “social avatar*” OR “social robot*” OR “social communicative machine*” OR “socially agent*” OR “socially assist*” OR “socially avatar*” OR “socially robot*” OR “socially communicative machine*” OR “speech activated interaction*” OR “speech enabled interaction*” OR “speech generated interaction*” OR “speech-activated interaction*” OR “speech-enabled interaction*” OR “speech-generated interaction*” OR “spoken dialogue agent*” OR “spoken dialogue assist*” OR “spoken dialogue system*” OR “talking bot*” OR “talking agent*” OR “talking avatar*” OR “talking robot*” OR “talking entit*” OR “talking assist*” OR “talking program*” OR “talking companion*” OR “talking chatbot*” OR

Domain	Search string
	“talking app*” OR “talking system*” OR “talking partner*” OR “talk bot*” OR “talk agent*” OR “talk avatar” OR “talk robot*” OR “talk entit*” OR “talk assist*” OR “talk program*” OR “talk companion*” OR “talk chatbot*” OR “talk app*” OR “talk system*” OR “talk partner*” OR “talkative bot*” OR “talkative agent*” OR “talkative avatar” OR “talkative robot*” OR “talkative entit*” OR “talkative assist*” OR “talkative program*” OR “talkative companion*” OR “talkative chatbot*” OR “talkative app*” OR “talkative system*” OR “talkative partner*” OR “text-based dialogue system*” OR “text-based healthcare chatbot*” OR “text-based synchronous chat*” OR “text based dialogue system*” OR “text based healthcare chatbot*” OR “text based synchronous chat*” OR “virtual chat*” OR “virtual chat agent*” OR “virtual chat expert*” OR “virtual chat entit*” OR “virtual chat assist*” OR “virtual chat app*” OR “virtual chat program*” OR “virtual chat companion*” OR “virtual chat bot*” OR “virtual chat robot*” OR “virtual chat avatar*” OR “virtual chat system*” OR “virtual chat partner*” OR “virtual conversational agent*” OR “virtual conversational bot*” OR “virtual conversational avatar” OR “virtual conversational robot*” OR “virtual conversational entit*” OR “virtual conversational assist*” OR “virtual conversational program*” OR “virtual conversational companion*” OR “virtual conversational chatbot*” OR “virtual conversational app*” OR “virtual conversational system*” OR “virtual conversational partner*” OR “virtual conversation agent*” OR “virtual conversation bot*” OR “virtual conversation avatar*” OR “virtual conversation robot*” OR “virtual conversation entit*” OR “virtual conversation assist*” OR “virtual conversation program*” OR “virtual conversation companion*” OR “virtual conversation chatbot*” OR “virtual conversation app*” OR “virtual conversation system*” OR “virtual conversation partner*” OR “virtual human interaction*” OR “voice-activated interaction*” OR “voice-driven interaction*” OR “voice-enabled interaction*” OR “voice-generated interaction*” OR “voice activated interaction*” OR “voice driven interaction*” OR “voice enabled interaction*” OR “voice generated interaction*” OR “vocal bot*” OR “vocal agent*” OR “vocal avatar*” OR “vocal robot*” OR “vocal entit*” OR “vocal assist*” OR “vocal program*” OR “vocal companion*” OR “vocal chatbot*” OR “vocal app*” OR “vocal system*” OR “vocal partner*” OR “vocal-based healthcare chatbot*” OR “alexa” OR “cORtana” OR “google home” OR “google assistant” OR “siri” OR “robotic psychological assist*” OR “smart virtual assist*” OR “smartbot*” OR “utterance avatar” OR “utterance robot*” OR “utterance bot*” OR “utterance agent*” OR “utterance entit*” OR “utterance assist*” OR “utterance program*” OR “utterance companion*” OR “utterance chatbot*” OR “utterance app*” OR “utterance system*” OR “utterance partner*”) AND
K2 (health and well-being)	(“illness” OR “ailment” OR “disab*” OR “disease*” OR “disORder*” OR “e-health” OR “ehealth” OR “health*” OR “m-health” OR “mhealth” OR “sick*” OR “wellbeing” OR “well-being”)
K3	NOT (“alexa-fluor” AND “alexa fluor”)

Google Scholar search query

Filters: from 2020, without patents and citations, sorted by relevance. Search performed on 19th November 2020.

("conversational agent*" OR "chatbot*" OR "conversation agent*" OR "conversation robot*" OR "conversational user-Interface*" OR "embodied conversational agent*") AND health
