# Supplementary material for: Conversational Agents for Health and Well-being Across the Life Course: Protocol for an Evidence Map
Source: JMIR Res Protoc. 2021 Sep 17;10(9):e26680. doi: 10.2196/26680 (PMC8486996; doi:10.2196/26680)
Supplement: Multimedia Appendix 2 [file resprot_v10i9e26680_app2.pdf]

Preliminary data extraction form for primary studies

What is the nature of literature on conversational agents in health and well-being?	**Article information**	**Information source**	Article identifier (DOI)
			Source name (e.g. Journal name, conference name)
			Publication type
			Year of publication
			Article title
		**Research group**	First author
			Affiliation of the first author
			Country
			Name of the corresponding author
			Corresponding author's email
			Funding
		**Study characteristic**	Design
			Aim
What are the characteristics of health interventions based on conversational agents?	**Health intervention**	**Setting**	
		**Target population**	Life course of the primary end-user
			Mean age of primary end-users
			Needs
			If a condition or complex needs are targeted, specify
			Additional end-users
			If yes, specify additional end-users
		**Intervention design**	Use of a model/framework/theory (if yes, identify)
		**Intervention type**	Health assessment
			Health education
			Social Support
			Behaviour change
			Other
		**Intervention target**	Eating
			Physical Activity
			Sleep and rest
			Hygiene
			Oral Hygiene
			Sexual and reproductive behaviour
			Breastfeeding
			UV radiation exposure
			Parenting
			Medication Adherence
			Health symptoms
			Managing symptoms
			Other
		**Intervention means**	Information provision
			Behaviour change technique (e.g. reminder, goal-setting, self-monitoring of behaviour or outcomes of behaviour)
			Other
		**External human intervention**	
		**Intervention duration**	
		**Intervention frequency**	
What are the characteristics of the automated conversations employed in these health interventions?	**Conversation**	**Interaction - Input**	Direct user input - Voice
			Direct user input - Written language (free text or pre-defined options)
			Direct user input - Still images
			Connection with wearables
			Vision
		**Interaction - Output**	Text and/or voice
			Non-language modalities
			Images
		**Dialogue engine**	Input processing (NL)
			Artificial intelligence
			Sentiment detection
			Emotions recognition based on facial expressions
			Knowledge needed
What are the characteristics of the agents employed in these health interventions?	**Agent**	**Embodiment**	Physical
			Virtual
			Physical
			No embodiment (e.g. text box)
		**Emotions**	
		**Role**	
		**Personality**	
		**Proactivity**	
		**Delivery channel**	
