# Supplementary material for: Conversational Agents for Health and Well-being Across the Life Course: Protocol for an Evidence Map
Source: JMIR Res Protoc. 2021 Sep 17;10(9):e26680. doi: 10.2196/26680 (PMC8486996; doi:10.2196/26680)
Supplement: Multimedia Appendix 3 [file resprot_v10i9e26680_app3.pdf]

Operational definitions

Term	Definition
Agent	Something that acts in an environment (Poole & Mackworth, 2017).
Anthropomorphic	Possessing or existing in a human bodily form.
Artificial intelligence	Field that studies the synthesis and analysis of computational agents that act intelligently, that is, it does what is appropriate for its circumstances and its goals; it is flexible to changing environments and changing goals; it learns from experience; and it makes appropriate choices given its perceptual and computational limitations (adapted from Poole & Mackworth, 2017).
Conversation | language modalities	Communication through the use of words (spoken or written) or signs, following rules of grammar.
Conversation | non-language modalities	Communication through elements such as facial expressions, gestures, paralinguistics, body language and posture, proxemics, eye gaze and appearance.
Conversational agent	Computer program designed to simulate human conversation using language (speech or text), potentially supplemented with non-language modalities.
Chatbot	Computer program designed to simulate human verbal or written conversations, which does not resort to non-language modalities.
Dialogue engine	System component that controls the dialogue flow.
E-coach	System that collects and processes a user's data in order to provide a personalized intervention able to support and motivate the user to reach a goal (El Kamali et al, 2020).
Embodied agent	Existing in a bodily form, such as a human body or an animal body, tangible or virtual.
Intervention (health)	Act performed for, with or on behalf of a person or population whose purpose is to assess, improve, maintain, promote or modify health, functioning or health conditions. (https://www.who.int/classifications/ichi/en/)
Intelligent agent	Autonomous entity that perceives its environment, reasons, and acts upon it in order to achieve some goal.
Intelligent virtual agent (IVA)	Autonomous, animated system that supports face-to-face interaction with people. It should incorporate some dimension of what can be considered intelligent behavior (for instance, advanced language processing or reasoning mechanisms, machine learning, emotion expressing) (de Antonio et al., 2001).
Learning agent	Agent that changes its behavior according to its past experience, incorporating it in its decision processes.
Machine learning	A type of artificial intelligence, which allows for broadening of the computer system capacity through its learning from data without being explicitly programmed (Tudor Car et al, 2020).
Meta-rule	A rule that defines how other rules, or sets of rules, should be used.
Natural language processing (or natural language understanding or interpretation)	The process whereby the machine translates human commands into a form in which the computer can understand, process, and revert to the user (Tudor Car et al, 2020).
Pattern-based	Based on the identification of textual patterns in the user input.

Term	Definition
Proactivity	The ability to provide adequate information or execute an appropriate action without being explicitly asked.
Relational agent	Computational artifacts designed to build and maintain long-term, social-emotional relationships with their users (Bickmore & Picard, 2005).
Rule-based (with or without meta-rules)	Following a predefined set of rules that determine actions/responses depending on a set of conditions.
Sentiment detection	Process of detecting positive, negative or neutral sentiments in text.
Script-based	Following a predefined sequence of steps that depend on patterns found on user input.
Virtual	Without physical existence, not tangible.
Virtual Assistant	Agent without physical existence that has the function of assisting the user in a specific task.

Bickmore, T. W., & Picard, R. W. (2005). Establishing and maintaining long-term human-computer relationships. *ACM Transactions on Computer-Human Interaction*, *12*(2), 293–327. https://doi.org/10.1145/1067860.1067867

de Antonio, A., Aylett, R., & Ballin, D. (Eds.). (2001). *Intelligent Virtual Agents: Third International Workshop, IVA 2001 Madrid, Spain, September 10–11, 2001 Proceedings* (Vol. 2190). Springer Berlin Heidelberg. https://doi.org/10.1007/3-540-44812-8

El Kamali, M., Angelini, L., Caon, M., Khaled, O. A., Mugellini, E., Dulack, N., Chamberlin, P., Craig, C., & Andreoni, G. (2020). NESTORE: Mobile Chatbot and Tangible Vocal Assistant to Support Older Adults' Wellbeing. *Proceedings of the 2nd Conference on Conversational User Interfaces*, 1–3. https://doi.org/10.1145/3405755.3406167

Poole, D., & Mackworth, A. (2017). *Artificial Intelligence: Foundations of Computational Agents* (2nd ed.). Cambridge University Press. http://artint.info/2e/html/ArtInt2e.html

Tudor Car, L., Dhinagaran, D. A., Kyaw, B. M., Kowatsch, T., Joty, S., Theng, Y.-L., & Atun, R. (2020). Conversational Agents in Health Care: Scoping Review and Conceptual Analysis. *Journal of Medical Internet Research*, *22*(8), e17158. https://doi.org/10.2196/17158
